# Retrospective Monocentric Analysis of Carmustine Wafer Implantation in Recurrent Glioblastoma: Impact on Survival and Key Prognostic Factors

**DOI:** 10.3390/curroncol33050238

**Published:** 2026-04-22

**Authors:** Naomi Houedjissin, Franziska Staub-Bartelt, Michael Sabel, Julia Steinmann, Hannah Fischer, Marion Rapp

**Affiliations:** 1 Department of Neurosurgery, University Hospital Düsseldorf, 40225 Düsseldorf, Germany; 2 Faculty of Medicine, Heinrich-Heine University, 40225 Düsseldorf, Germany; 3 Department of Neurosurgery, University Hospital Giessen, 35392 Giessen, Germany; 4 Brain Cancer Center, Beta Clinic, 53127 Bonn, Germany; 5 Department of Neurosurgery, Sana Clinic, 47055 Duisburg, Germany; 6 Evangelisches Klinikum Bethel, 33615 Bielefeld, Germany

**Keywords:** glioblastoma, recurrence, local chemotherapy, carmustine wafer, high-grade glioma

## Abstract

Objective: The implantation of biodegradable carmustine (BCNU) wafers is a treatment option for recurrent high-grade glioma (HGG), but its efficacy is debated. We evaluated its impact on overall survival (OS) and survival after recurrence (SAR) considering recurrence timing after first-line treatment. Methods: In this single-center retrospective study, we analyzed patients who underwent surgery for glioblastoma (GBM) recurrence following initial diagnosis (pre- and post-WHO classification 2016) between 2007 and 2022. All patients received standard first-line therapy, including maximal safe resection, radiotherapy with concomitant temozolomide, and adjuvant temozolomide. Recurrent GBM treatment involves resection, with or without adjuvant chemo- and/or radiotherapy. Patients who received carmustine wafer implantation (CWI) during resection were compared to those without wafer placement. Recurrences were classified by timing relative to first-line therapy: (1) post-radiochemotherapy, pre-adjuvant temozolomide; (2) during adjuvant temozolomide; (3) during prolonged temozolomide; (4) >1 month after completion of all therapy. Primary endpoints were OS and SAR, with prognostic factors analyzed. Results: A total of 176 patients were enrolled, with 59.7% (105/176) receiving CWI. Recurrence treatment included surgery without adjuvant therapy in 23.3% (41/176) of cases (26.7% of CWI+ and 18.3% of CWI−), adjuvant chemotherapy in 39.8% (70/176) (41% of CWI+ and 38% of CWI−), radiotherapy in 7.4% (13/176) (7.6% of CWI+ and 7% of CWI−), and combined radiochemotherapy in 29.5% (52/176) (24.8% of CWI+ and 36.6% of CWI−). No significant differences were found between groups in age (*p* = 0.684), residual tumor volume after initial (*p* = 0.988) or recurrence surgery (*p* = 0.356), MGMT status (*p* = 0.766) and KPS post 1st-line-therapy (*p* = 0.833). Median OS was 20 months [range 18–24] for CWI+ and 22 months [range 20–27] for CWI− (*p* = 0.487). The median SAR was 10 months [range 8–12] for CWI+ and 12 months [range 10–13] for CWI− (*p* = 0.252). Later recurrence (type 4) significantly correlated with prolonged OS (HR 0.16, 95% CI: 0.04–0.66, *p* = 0.011). Age (*p* < 0.001), MGMT methylation (*p* = 0.017), and smaller residual tumor volume post-recurrence surgery (*p* = 0.008) were also associated with longer survival. Conclusions: CWI did not significantly improve OS or SAR in recurrent GBM patients. However, younger age, MGMT methylation, smaller residual tumor volume, and later recurrence were linked to better survival outcomes, underscoring their prognostic importance.

## Simple Summary

Glioblastoma is the most aggressive form of brain cancer in adults, and despite current treatment approaches, it still cannot be cured. When the tumor grows again, doctors may perform another operation and sometimes place small biodegradable wafers containing a cancer drug directly into the space left by the tumor. We studied 176 people with returning glioblastoma to find out whether this local treatment helped patients live longer. We compared patients who received these drug-containing wafers during surgery with patients who did not. We found that the wafers did not improve overall survival and did not extend survival after the cancer came back. However, some factors were linked to better outcomes: younger age, a tumor with a more favorable biological profile, a smaller amount of tumor left after repeat surgery, and a later return of the disease. These findings suggest that this wafer treatment may not provide an additional benefit in modern care for returning glioblastoma. This is valuable for patients and doctors because it can support more informed treatment decisions.

## 1. Introduction

Diffuse gliomas comprise 70% of the most common malignant brain tumors in adults, with glioblastoma representing the most aggressive subtype, accounting for approximately 50% of them [1]. The incidence of glioblastoma continues to rise, with an estimated annual rate of 5 per 100,000 individuals [2]. 

While a standard first-line treatment protocol has been established for glioblastoma, there remains no universally accepted strategy for managing tumor recurrence. According to the current WHO classification, primary therapy of newly diagnosed IDH-wildtype glioblastoma (WHO Grade 4) [3] involves maximal safe and, if possible, supramarginal resection [4], followed by radiochemotherapy combined with tumor treating fields (TTF) [5,6,7]. With this aggressive treatment scheme, overall survival (OS) could be increased to 20.9 months [6], and in a subgroup of MGMT promoter methylated patients even to 48.1 months [7], with a 5-year survival rate up to 34% [6]. However, despite this huge success, treatment of glioblastoma patients remains palliative, and tumor recurrence is inevitable. In addition to surgery, adjuvant chemotherapy with temozolomide is a commonly used treatment option for recurrent glioblastoma. Despite such interventions, the median progression-free interval remains limited to 3–4 months until the second recurrence, and survival after recurrence (SAR) is approximately 9.5–10.5 months [8].

In recent years, the implantation of biodegradable carmustine wafers, also known as Gliadel^®^ wafers, has been revisited as a potential local therapeutic option for recurrent glioblastoma. Carmustine wafers consist of 1,3-bis(2-chloroethyl)-1-nitrosourea (BCNU) embedded in a biodegradable polyanhydride copolymer matrix that undergoes hydrolytic degradation upon contact with water, releasing BCNU into the surrounding brain tissue [9] in high-dose concentrations with a depth of 1–3 mm and low-dose concentrations extending up to 1–2 cm from the implant [10,11]. The release begins within hours, peaking within the first few days, with most of the drug being released over approximately 5 days [9]. Complete degradation typically occurs within 6 to 8 weeks, although degradation can be delayed due to poor fluid contact or trapped air in the resection cavity [12].

Despite the initial promise that interstitial BCNU chemotherapy could be an effective treatment option for recurrent malignant gliomas [13], recent clinical studies evaluating the efficacy of carmustine wafers have yielded heterogeneous results: While some have demonstrated a significant OS benefit for CW implantation at initial diagnosis of HGG and at recurrent HGG [14,15,16,17], others found a prolongation in progression-free survival (PFS) without a corresponding improvement in OAS [18,19]. 

Routine use has declined, for example in France, where a significant decrease in implantation rates has been observed [20]. The main concerns include uncertain efficacy and the risk of perioperative complications, such as cerebral oedema, convulsion, impaired healing and surgical site infection, fever and lymphocytopenia, cerebrospinal fluid (CSF) leakage, and meningitis [15,18,19,20,21,22]. A causal relationship can be suggested with further complications in 30% of cases with infections among patients with impaired healing, and approximately 20% of patients developed convulsions among patients with cerebral oedema [21]. Although postoperative complications associated with CWI are well documented (surgical site infection (SSI), CSF leakage, meningitis), their direct impact on OS has not been consistently demonstrated [18,20]. 

Given the conflicting evidence regarding the clinical utility of carmustine wafers, there is a growing consensus that more refined patient selection criteria are needed. Outcomes appear to vary significantly depending on prognostic factors, such as age (especially >65 years), extent of resection, and the use of concomitant radiotherapy [14,20,23]. Also, preoperative clinical and laboratory parameters may help identify patients who are most likely to benefit from local BCNU therapy [24,25]. However, it remains unclear whether specific patient subgroups, based on these or other clinical parameters, derive a distinct survival benefit from carmustine wafer implantation.

Therefore, we herein evaluated its impact on overall survival (OS) and survival after recurrence (SAR), considering the recurrence timing after first-line treatment.

## 2. Methods

### 2.1. Patients and Standard of Care

In this single-center retrospective study, we identified 176 patients diagnosed with glioblastoma (GBM) who met the following inclusion criteria: all patients received standard first-line therapy, including maximal safe surgical resection followed by radiotherapy with concomitant temozolomide and subsequent adjuvant temozolomide to reduce treatment-era heterogeneity. In addition, only patients with a histopathologically confirmed first recurrence after surgery were included in the study.

At first recurrence, patients underwent additional surgical resection followed by adjuvant chemotherapy and/or radiotherapy. Treatment strategies at recurrence were not fully standardized and could vary according to the individual clinical setting. Patients who received carmustine wafer implantation (CWI) during re-resection were compared to those who did not receive CWI. Post-recurrence treatment variables were systematically recorded and considered in the statistical evaluation.

Initial diagnoses (before and after the implementation of the 2016 and 2021 WHO classifications) were performed between 2007 and 2022 at our neuro-oncology department at the University Hospital Düsseldorf.

Following initiation of standard therapy, patients underwent MRI scans every three months. Imaging was performed at the Institute of Radiology, University Hospital Düsseldorf, using a Magnetom Skyra (Siemens Healthiness, Erlangen, Germany). Volumetric analysis was conducted to assess residual tumor volume. These volumetric data are reported in Table 1 and were considered in the survival analyses. If a new contrast-enhancing lesion appeared within 12 weeks after the end of radiotherapy, treatment was continued, and a follow-up MRI was performed 6 to 8 weeks later to confirm or rule out progression, in accordance with the RANO criteria [26]. However, in cases of clear clinical deterioration highly suggestive of tumor progression (and not attributable to corticosteroid reduction), further treatment decisions were made based on the patient’s clinical presentation [27]. Progressive disease (PD) on MRI, as defined by the RANO criteria, included an increase of equal to or more than 25% in the perpendicular diameters of enhancing lesions in total, the progression of nonmeasurable lesions, or the appearance of new lesions, which were not assignable to a decrease in corticosteroid dosage [27]. With the introduction of the updated RANO 2.0 criteria, assessment of non-enhancing tumor progression is optional but no longer mandatory for glioblastoma [28]. 

### 2.2. Surgical Treatment and Carmustine Wafer Implantation

Upon radiological confirmation of PD via MRI as the primary imaging modality, the decision for resection with potential carmustine wafer implantation, adjuvant (radio-) chemotherapy, or initiating best-supportive care was deliberated. Additionally, PET imaging was not performed routinely but was used selectively in cases with inconclusive MRI findings, particularly when progression could not be reliably distinguished from treatment-related changes.

Although the decision for implantation of a carmustine wafer was based on individual clinical and surgical considerations, including tumor localization, extent of resection, and the patient’s general condition, specific intraoperative reasons for omitting wafer placement, such as suspected cerebrospinal fluid space communication, local infection risk, or deep tumor location, were not documented in a sufficiently standardized manner for retrospective evaluation. It did not adhere to a standardised allocation. Following partial or complete re-resection, 4–5 carmustine wafers were placed into the resection cavity in eligible patients. 

### 2.3. Neuropathology

Histopathological examination was performed following either partial or complete open surgical resections. Diagnoses were established using formalin-fixed and paraffin-embedded tissue samples and classified according to either the 2007 or 2016 WHO classification system [3,29,30,31]. Only patients with histologically confirmed recurrent glioblastoma were included in the study. 

### 2.4. Data Collection, Definition and Endpoints

Clinical and demographic data were extracted from medical records, including age, sex, Karnofsky Performance Status (KPS), and MGMT promoter methylation status. Imaging data includes tumor location and postoperative residual volume after initial and recurrent surgeries. Treatment-related variables include the use of chemotherapy, radiotherapy, bevacizumab, and tumor-treating fields (TTF). In alignment with Perry et al. [8], recurrences were classified by timing relative to first-line therapy: (1) post-radiochemotherapy, prior to adjuvant temozolomide, (2) during adjuvant temozolomide, (3) during extended temozolomide therapy and (4) more than 1 month after completion of temozolomide treatment [8]. These categories were chosen to reflect clinically distinct patterns of recurrence timing in relation to ongoing or completed first-line therapy and to differentiate earlier relapse during active treatment from later relapse after treatment completion. The study intended to evaluate the effect of carmustine wafer implantation on survival outcomes in recurrent GBM compared to standard treatment without CWI. In both groups, patients received further adjuvant treatment. Two primary endpoints were specified (1) overall survival (OS) and (2) survival after recurrence (SAR). OS is defined as the time from the initial MRI indicating GBM (before histopathological confirmation) to death. SAR is defined as the time from the MRI indicating the first recurrence (before histopathological confirmation) to death.

### 2.5. Statistical Analysis and Ethical Considerations

The normal distribution of the data was assessed using the Shapiro–Wilk test. Descriptive statistics include means and standard deviations, medians, ranges, and percentages. Data visualization and presentation were achieved using tables and graphs. Survival outcomes were assessed using Kaplan–Meier estimates for OS and SAR [32,33]. Multivariable Survival Analyses were employed to identify prognostic factors associated with OS and SAR [34], with a forest plot showing hazard ratios (HR), 95% confidence intervals (CI), and *p*-values [35,36,37,38]. Due to the retrospective nature of the study, no stratified randomization or blinding was performed. Statistical analyses were conducted using Jamovi software (The jamovi project (2025). jamovi (Version 2.6) [39,40]. The study was approved by the Ethics Committee of Heinrich Heine University Düsseldorf (approval date 14th February 2022, Study No. 2021-1743).

## 3. Results

### 3.1. Patient Population and Treatment-Related Characteristics

A total of 176 patients met the inclusion criteria for this study. Among them, 105 patients (59.7%) underwent carmustine wafer implantation during surgery for first recurrence. Baseline clinical and demographic data for the full cohort and for both treatment groups are provided in Table 1.

Baseline characteristics were broadly comparable between patients with and without CWI. Age at initial diagnosis was nearly identical in both cohorts (57.9 vs. 57.2 years; *p* = 0.684) with a mean age of 57.6 years (SD 10.8). Although the proportion of male patients was higher in the CWI+ group, this difference did not reach statistical significance (70.5% vs. 56.3%; *p* = 0.054). Functional status at recurrence, as measured by Karnofsky Performance Status (KPS), showed no relevant imbalance between groups (*p* = 0.833). The distribution of MGMT promoter methylation status was likewise comparable, with methylation identified in 33.1% of the overall cohort (*p* = 0.766).

Residual postoperative tumor volume after both primary and recurrence surgery was similar between groups, with no statistically significant differences observed for either time point (*p* = 0.988 and *p* = 0.356). In addition, the use of further treatment after recurrence, including chemotherapy (*p* = 0.207), radiotherapy (*p* = 0.128), bevacizumab (*p* = 0.923), and tumor-treating fields (*p* = 0.987), was not significantly different between the two cohorts.

### 3.2. Survival Analysis

**Figure 1:**
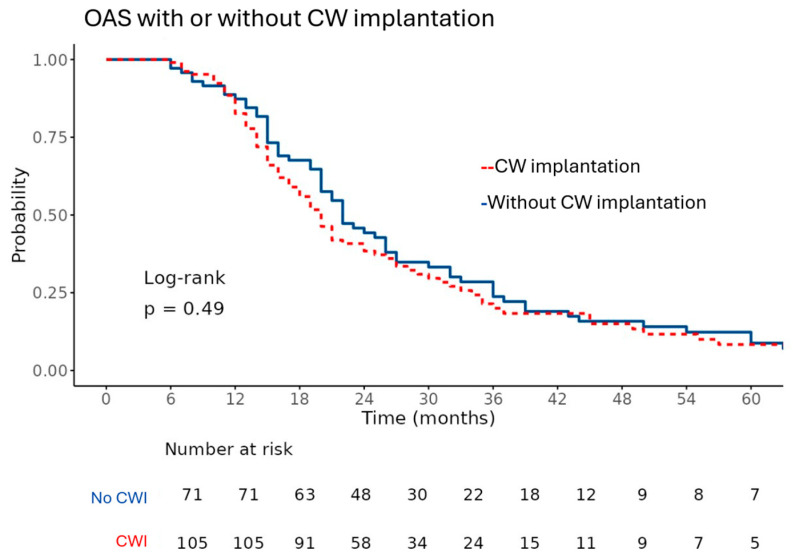
Kaplan–Meier survival curves comparing overall survival (OAS) in patients with and without carmustine wafer (CW) implantation show similar survival probabilities over time. The x-axis represents time in months, and the y-axis represents survival probabilities ranging from 0.00 to 1.00.

**Figure 2:**
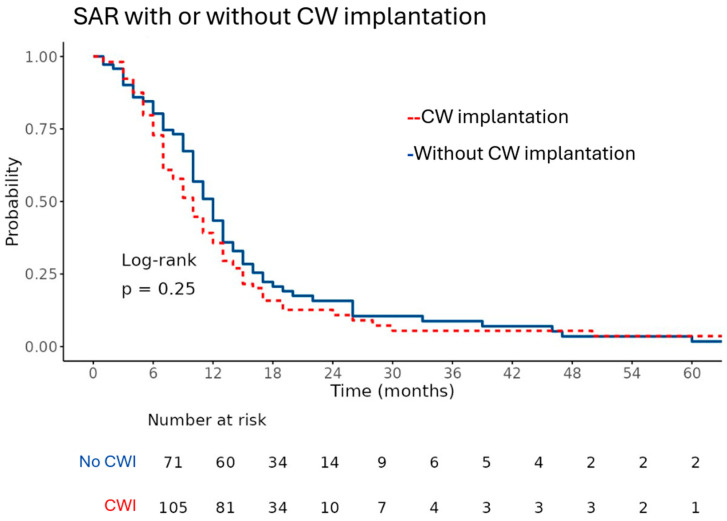
Kaplan–Meier survival curves comparing survival after recurrence (SAR) in patients with and without carmustine wafer (CW+/CW−) implantation show similar survival probabilities over time. The x-axis represents time in months, and the y-axis represents survival probabilities ranging from 0.00 to 1.00.

We evaluated OS and SAR using Kaplan–Meier methodology, with the corresponding curves shown in Figure 1 and Figure 2. Patients treated with CWI had a median overall survival of 20 months (95% confidence interval 18–24), compared with 22 months (95% confidence interval 20–27) in patients without wafer implantation. This difference was not statistically significant (*p* = 0.487), suggesting that wafer placement at first recurrence was not associated with a measurable survival advantage in this cohort.

Similarly, the median SAR was 10 months [95% CI: 8–12] in the CWI+ group and 12 months [95% CI: 10–13] in the CWI– group, which was also not statistically significant (*p* = 0.252). The survival curves for both outcomes closely overlapped throughout the follow-up period, reflecting broadly comparable survival probabilities across the groups. 

Subgroup analyses were conducted based on four predefined recurrence intervals depending on the recurrence time-point, as mentioned above. No statistically significant differences in OS were identified in any subgroup, as shown in Table 2.

### 3.3. Predicting Factors: Multivariable Survival Analysis

To identify independent prognostic factors associated with survival, a Cox proportional hazards model using a complete-case approach was applied to a subset of 76 patients for whom complete data on key clinical covariates were available. Within this subset, 69 patients experienced a survival event (death). The model demonstrated strong predictive performance, with a concordance index of 0.780 (standard error = 0.027), reflecting good discrimination between higher- and lower-risk patients. The pseudo-R^2^ value was 0.539, indicating that over half of the variability in survival was accounted for by the included variables. The model was statistically significant overall (likelihood ratio test: χ^2^ = 58.773, degrees of freedom = 13, *p* < 0.001). 

**Figure 3:**
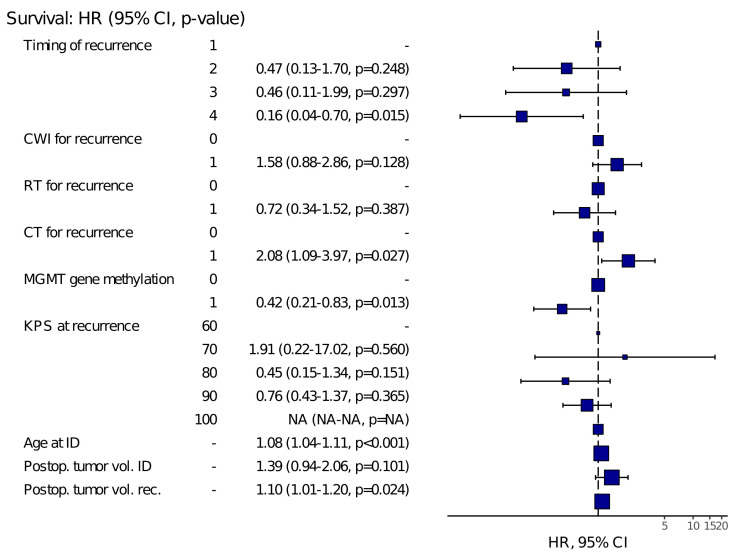
Forest plot showing hazard ratios (HR), 95% confidence intervals (CI), and *p*-values from multivariate analysis of factors affecting survival. Model Metrics: Survival: HR (95% CI, *p*-value) OAS Number in data frame = 76, Number in model = 76, Missing = 0, Number of events = 69, Concordance = 0.780 (SE = 0.027), R-squared = 0.539 (Max possible = 0.998), Likelihood ratio test = 58.773 (df = 13, *p* = 0.000).

Significant predictors include timing of recurrence type 4 (HR 0.16, *p* = 0.015), chemotherapy for recurrence (HR 2.08, *p* = 0.027), MGMT gene methylation (HR 0.42, *p* = 0.013), age at initial diagnosis (HR 1.08, *p* < 0.001), postoperative tumor volume at initial diagnosis (HR 1.39, *p* = 0.011), and postoperative tumor volume at recurrence (HR 1.10, *p* = 0.024). A summary of hazard ratios, confidence intervals, and p-values for each covariate is shown in the forest plot (Figure 3).

## 4. Discussion

### 4.1. Key Results

In this retrospective study, we evaluated the effect of carmustine wafer implantation (CWI) on survival outcomes in patients with recurrent glioblastoma (GBM). Our findings demonstrate that CWI did not significantly improve overall survival (OS) or survival after recurrence (SAR) compared to patients who did not receive wafers. Median OS in the CWI+ group was 20 months compared to 22 months in the CWI– group, while median SAR was 10 and 12 months, respectively. These findings persisted across subgroup analyses and multivariable models, suggesting that carmustine wafer implantation does not confer a significant survival advantage in the context of modern multimodal treatment regimens. Treatment and control groups were well matched across key clinical parameters, providing a balanced foundation for subsequent survival analyses. 

It is important to highlight that in our study, CWI was used exclusively at the first histopathological confirmed recurrence of GBM. The rationale for this approach lies in the absence of a clearly established standard therapy for recurrent disease. While treatment at initial diagnosis is well defined, most notably through the standard of care combining maximal safe resection with radiotherapy and concomitant/adjuvant temozolomide, there is no universally accepted regimen for recurrence. This clinical uncertainty motivated our investigation into whether the addition of localised chemotherapy via carmustine wafers might influence survival outcomes in the recurrent setting. 

### 4.2. Predicting Factors

Multivariate analysis in our study identified several factors independently associated with OS. Patients with recurrence type 4 (indicating late recurrence after first-line treatment completion) had significantly better outcomes, while those who received chemotherapy for recurrence had a higher hazard of death, possibly reflecting a selection for more aggressive or infiltrative disease. Therefore, the association between chemotherapy at recurrence and poorer survival should be interpreted with caution, as it may reflect confounding by indication. Patients selected for systemic treatment in a recurrent setting are likely to have had more aggressive or less favourable disease characteristics that were not fully captured in the available dataset. MGMT promoter methylation remained a strong, favourable prognostic marker, in line with existing literature. Other significant predictors included increased age at initial diagnosis, greater postoperative tumor volume following the initial surgery, and greater tumor volume at recurrence (HR = 1.10, *p* = 0.024). These results underscore the multifactorial nature of GBM prognosis and highlight the need for individualised treatment strategies based on molecular and volumetric parameters. 

### 4.3. Impact of Carmustine Wafer on Survival

Our results are in contrast with earlier studies that reported a survival advantage with CWI. In the pivotal trial by Brem et al., patients with recurrent glioma treated with carmustine wafers had a median survival of 7.8 months, compared to 5.3 months in the placebo group, representing a significant relative benefit in the pre-temozolomide era [13]. Similarly, Duntze et al. observed a median survival of 18 months in patients treated with carmustine wafers at first diagnosis [17]. These results, once considered promising, are notably lower than the median survival observed in both the CWI+ and CWI– groups in our cohort. The absence of a survival benefit in our data likely reflects substantial advancements in the overall management of glioblastoma over the past two decades. This interpretation is also in line with more recent literature. Although some newer reports still suggest potential benefit in selected malignant glioma cohorts [14], contemporary real-world series have emphasized heterogeneous outcomes and patient selection, and nationwide data indicate that routine wafer implantation has declined over time [20,23]. A recent nationwide propensity score-matched analysis in newly diagnosed high-grade glioma did not demonstrate an overall survival benefit associated with carmustine wafer implantation [41].

The establishment of standardised therapeutic protocols, the routine pursuit of supramarginal resection whenever feasible, and the incorporation of novel treatment modalities such as TTF have collectively led to markedly improved survival outcomes in patients with glioblastoma. These substantial advancements in multimodal management likely diminish the relative contribution of local chemotherapy, which once demonstrated modest benefit in earlier therapeutic contexts. Furthermore, refinements in microsurgical technique, enhanced neuroimaging enabling earlier detection of disease progression, and the broader application of intensive salvage therapies may further attenuate the incremental impact of local interventions such as CWI. 

Importantly, the potential risks associated with CWI must be considered when weighing its clinical utility. Bock et al. reported that among 44 patients treated with CWI followed by concomitant radiochemotherapy, 52% experienced adverse events and 43% experienced grade 3 or 4 toxicities, including cerebral oedema, wound healing abnormalities, CSF leakage, meningitis, and thromboembolic complications [22]. Consequently, treatment delays occurred in 18% of patients during radiochemotherapy and in 9% during subsequent temozolomide therapy. These safety concerns further challenge the rationale for routine use of carmustine wafers in the current therapeutic landscape, especially when no survival advantage is observed. 

### 4.4. Limitations

This study has several statistical and methodological limitations. First, its retrospective, single-centre design inherently limits the ability to control for all confounding variables and may introduce selection bias. The decision to implant carmustine wafers was non-randomised and clinician-dependent, potentially reflecting underlying clinical judgments not fully captured in the available data. This introduces a potential risk of selection bias, as factors influencing the decision for implantation may not have been fully captured in the available dataset. Although the treatment and control groups were well matched on key baseline parameters, unmeasured confounders may have influenced outcomes. Moreover, a notable limitation of this study is the long inclusion period spanning from 2007 to 2022, during which substantial advances in glioblastoma diagnostics and therapeutics occurred. The adoption of molecularly informed classifications, the introduction of novel systemic regimens such as the CeTeG/NOA-09 protocol, and the increasing use of TTF have markedly altered the standard of care and improved overall survival in contemporary cohorts. This temporal heterogeneity was partly reduced by restricting the cohort to patients who had all received a comparable first-line Stupp-based regimen after initial diagnosis. However, it may have introduced confounding effects when comparing outcomes across patients treated under different clinical paradigms. The management at recurrence was more heterogeneous, reflecting the lack of a uniformly established second-line standard. These treatment-related variables were recorded and compared between groups, but subgroup stratification by treatment era was limited by sample size constraints. While the single-center design may have reduced interinstitutional variability, the potential influence of evolving diagnostic and therapeutic standards still need to be considered when evaluating the survival findings. In addition, some surgically relevant details were not available in a sufficiently standardized form for all patients. In particular, the extent of resection could not be uniformly categorized retrospectively as gross total resection, subtotal resection, or biopsy; and specific intraoperative reasons for not using carmustine wafers were not consistently documented. Therefore, residual confounding related to surgical decision-making cannot be excluded.

Furthermore, while multivariable Cox regression was used to adjust for prognostic factors, the reduced sample size (*n* = 76) for complete-case multivariate analysis limits statistical power and generalizability. This subset represents only those cases with complete clinical, molecular, and volumetric datasets, resulting in exclusion of patients with missing parameters. The missing data mainly reflected the retrospective design and long inclusion period, including unavailable MGMT promoter methylation status in a subset of earlier cases, incomplete or non-standardized postoperative volumetric measurements, and inconsistent documentation of Karnofsky Performance Status in routine clinical records. While complete-case analysis ensures methodological transparency, it may limit statistical power and generalizability of the multivariate findings. Multiple imputation was not feasible due to the retrospective design and heterogeneity of missing variables. Nonetheless, the consistency of univariate and multivariable trends supports the internal validity of the observed associations, although results should be interpreted with caution. 

A further limitation of this study is the lack of a standardized retrospective assessment of postoperative complications. Although safety concerns such as infection, seizures, cerebral oedema, cerebrospinal fluid leakage, and wound-healing disorders are clinically relevant in the context of CWI, these events could not be assessed retrospectively in a sufficiently standardized manner to allow reliable comparative analysis. Lastly, there is an absence of extended molecular stratification beyond MGMT promoter methylation. Notably, IDH mutation status was not consistently available throughout the entire inclusion period (2007–2022), reflecting the historical nature of the cohort and the gradual incorporation of molecular diagnostics into routine glioma classification. Consequently, it was not possible to ensure a uniform distinction between IDH-wildtype glioblastoma and tumors that would currently be classified as grade 4 IDH-mutant astrocytoma for all cases. In addition, other relevant biomarkers, such as TERT promoter mutation, EGFR amplification, CDKN2A/B deletion, and ATRX status, have become central to the molecular classification of glioblastoma following the 2021 WHO update. However, due to the historical nature of part of our cohort and the retrospective data collection, these molecular parameters were not available for the majority of cases. Consequently, potential molecular heterogeneity within the study population could not be fully captured, and residual confounding by unmeasured genomic alterations cannot be excluded. Future prospective studies incorporating comprehensive molecular profiling will be essential to refine patient stratification and to better define subgroups that may benefit from local carmustine therapy. 

## 5. Conclusions

In conclusion, our findings suggest that in the context of modern glioblastoma treatment including advanced surgical, systemic, and supportive strategies, the incremental benefit of carmustine wafer implantation is no longer evident, even at first recurrence where no standard therapy is defined. While several factors were linked to more favorable survival outcomes, no subgroup with a clearly identifiable benefit from wafer implantation could be defined based on the available data.

While historically associated with improved outcomes, the role of CWI appears diminished when layered onto contemporary treatment protocols. Its routine use should be carefully reconsidered and possibly limited to specific clinical scenarios or investigational settings where its benefit can be more clearly defined.

## Figures and Tables

**Table 1:** Patient characteristics in carmustine wafer use and non-use groups.

Parameters	All Patients (*n* = 176)		Carmustine Wafer Implantation at 1st Recurrence			
	No.	%	Yes (*n* = 105)		No (*n* = 71)	
			No.	%	No.	%
Mean age at initial diag., y, (SD)	57.6	(10.8)	57.9	(10.3)	57.2	(11.6)
Gender, *n*						
male	114	64.8	74	70.5	40	56.3
female	62	35.2	31	29.5	31	43.7
MGMT gene status (missing: 40)						
unmethylated	91	66.9	55	67.9	36	65.5
methylated	45	33.1	26	32.1	19	34.5
KPS at initial diag., *n* (missing: 80)						
100	37	38.5	21	36.2	16	42.1
90	47	49	30	51.7	17	44.7
80	8	8.3	4	7	4	10.6
70	3	3.1	2	3.4	1	2.6
<70	1	1	1	1.7	0	0
KPS before therapy for recurrence, *n* (missing: 13)						
100	47	28.8	28	28.3	19	29.7
90	84	51.5	53	53.5	31	48.4
80	21	12.9	12	12.1	9	14.1
70	10	6.1	5	5.1	5	7.8
<70	1	0.6	1	1	0	0
Postoperative tumor vol. initial diag.: Mean, ml, (SD)/missing: 62	0.47	(1.21)	0.439	(1.09)	0.517	(1.38)
Postoperative tumor vol. recurrence: Mean, ml, (SD)/missing: 22	1.065	(2.49)	1.279	(2.95)	0.755	(1.56)
Treatments for recurrence						
Chemotherapy						
Yes	122	69.3	69	65.7	53	74.6
No	54	30.7	36	34.3	18	25.4
Radiotherapy						
Yes	65	36.9	34	32.4	31	43.7
No	111	63.1	71	67.6	40	56.3
Bevacizumab						
Yes	12	6.8	7	6.7	5	7
No	164	93.2	98	93.3	66	93
Tumor-treating fields						
Yes	5	2.8	3	2.9	2	2.8
No	171	97.2	102	97.1	69	97.2

**Table 2:** Subgroup survival analyses for OAS depending on the recurrence time-point.

	Carmustine Wafer Implantation at 1st Recurrence						Test		
	Yes (*n* = 105)			No. (*n* = 71)			Log-Rank		
	*N*	Censored	Median [Lower, Up.] in Months	*N*	Censored	Median [Lower, Up.] in Months	x^2^	df	*p*
Recurrence Timing 1	7	0	10 [7, NaN]	6	1	11.5 [8, NaN]	0.528	1	0.467
Recurrence Timing 2	48	10	15 [14, 19]	32	1	18 [15, 22]	0.223	1	0.637
Recurrence Timing 3	9	1	20 [18, NaN]	4	0	17 [14, NaN]	0.506	1	0.477
Recurrence Timing 4	41	9	32 [27, 45]	27	3	36 [32, 60]	1.10	1	0.295

## Data Availability

The data presented in this study are available from the corresponding author on reasonable request. They are not publicly available due to legal, ethical, and privacy restrictions.
